# Competent blastocyst and receptivity endometrium improved clinical pregnancy in fresh embryo transfer cycles: a retrospective cohort study

**DOI:** 10.1186/s12884-024-06399-x

**Published:** 2024-04-11

**Authors:** Longmei Wang, Pingping Qiu, Lizhi Jiang, Ping Li, Yufei Jiang

**Affiliations:** Xiamen Key Laboratory of Reproduction and Genetics, Department of Reproductive Medicine, Women and Children’s Hospital, School of Medicine, Xiamen University, Xiamen, 361003 Fujian China

**Keywords:** Available blastocyst formation rate, Receptivity endometrium, Day 3 or day 5, Fresh embryo transfer, Clinical pregnancy

## Abstract

**Background:**

Embryo quality is usually regarded as a key predictor of successful implantation and clinical pregnancy potential. The identification of embryos that have the capacity to implant and result in a healthy pregnancy is a crucial part of in vitro fertilization (IVF). Usually, morphologically high-quality embryos are chosen for embryo transfer in IVF treatment. The aim of this study was to assess the association between the available blastocyst formation rate and the clinical pregnancy outcome following the first fresh embryo transfer cycle and provide systematic individual treatment to adjust endometrial receptivity for the next transfer cycle.

**Methods:**

This retrospective, single-center study included 512 fresh embryo transfers conducted between 11/2019 and 08/2021, which consisted of 385 cleavage-stage (Day 3) and 127 blastocyst-stage (Day 5) embryo transfers. The two groups were divided into a clinical pregnancy group and a nonclinical pregnancy group for comparison. The association between the available blastocyst formation rate and the clinical pregnancy rate in the Day 3 and Day 5 transfer groups were considered.

**Results:**

In the Day 3 group, there were 275 clinical pregnancies, and the clinical pregnancy rate was 71.43%. Although the two pronuclei (2PN) oocyte rate and available embryo rate at Day 3 were significantly higher in the clinical pregnancy group than the nonclinical pregnancy group (*P* < 0.05), the blastocyst formation rate and the available blastocyst formation rate were not significantly different between the clinical pregnancy group and the nonclinical pregnancy group (*P* > 0.05). In the Day 5 group, there were 81 clinical pregnancies, and the clinical pregnancy rate was 63.78%. No baseline characteristics showed any obvious differences between the clinical pregnancy group and nonclinical pregnancy group (*P* > 0.05). The blastocyst formation rate in the nonclinical pregnancy group was higher than that in the clinical pregnancy group, but the difference was not statistically significant (81.06% vs. 77.03%, *P* = 0.083). Interestingly, the available blastocyst formation rate and the Day 5 available blastocyst formation rate were significantly higher in the nonclinical pregnancy group than the clinical pregnancy group (66.19% vs. 60.79%, *P* = 0.014; 54.58% vs. 46.98%, *P* = 0.007).

**Conclusions:**

In fresh cycles, the available blastocyst formation rate was not associated with the clinical pregnancy outcome for Day 3 embryo transfers, and the available blastocyst formation rate was not positively correlated with the clinical pregnancy outcome for Day 5 embryo transfers.

**Supplementary Information:**

The online version contains supplementary material available at 10.1186/s12884-024-06399-x.

## Introduction

Along with the development of assisted reproductive technology (ART), there has been a significant improvement in successful pregnancies [1]. Embryos created with assisted reproductive technology (ART, or IVF) can be transferred into a woman’s uterus at either the cleavage (Day 3) or the blastocyst stage (Days 5–7). Advances in embryo culture up to the blastocyst stage enable a better selection of embryos with a superior developmental capacity and consequently a higher implantation potential [2, 3]. The rationale for blastocyst transfer is to improve both uterine and embryonic synchronicity and enable self-selection of available embryos, thus resulting in better live birth rates [4]. Embryo transfer at the blastocyst stage increases the clinical pregnancy rate per embryo transferred, and this is especially important in the context of single embryo transfer (SET) policies, intending to reduce multiple gestations [5, 6]. However, it is possible that the culture of embryos to the blastocyst stage in the laboratory leads to the loss of some embryos that may have survived inside the uterus. Thus, at many IVF centers, cleavage-stage transfers are performed in patients with few available embryos to reduce the incidence of cycle cancellation if no embryo reaches the blastocyst stage, and Day 3 fresh transfer is also recommended for women with previous failed blastocyst transfers [7]. Most notably, blastocyst-stage transfer does not appear to increase the cumulative live birth rate (CLBR) compared with cleavage-stage transfer [8].

The blastocyst participates in the first physical and physiological interaction with the maternal endometrium to initiate implantation, which is a complex process involving both the blastocyst and the maternal endometrium, which is receptive for 48 h 7–10 days after ovulation [9]. Interactions between the uterus and the blastocyst can only occur during a limited defined period, known as the “window of implantation” (WOI), during which the maternal endometrium undergoes dramatic changes [10]. Successful implantation requires a receptive endometrium, a functional embryo and a synchronized dialog between them, disorders in these communications are tightly associated with adverse pregnancy outcomes [9, 11]. The ability of the endometrium to allow normal implantation is termed receptivity, and optimal receptivity leads to a normal implantation process that serves as the foundation for a healthy pregnancy [12]. However, luteal phase defects and a lack of synchrony in the development of different cellular compartments of the endometrium could decrease embryo implantation synchronization.

Although the live birth rate per transfer cycle is generally used as a measure of the ART treatment outcome, it is not a good indicator of the biological efficacy of oocytes or embryos. Culture to the blastocyst stage can further eliminate some embryos with chromosomal abnormalities or no development potential, so we believe that the available blastocyst formation rate may be used to more truly and accurately assess the development potential of oocytes and embryos. Therefore, we aimed to investigate whether the available blastocyst formation rate could be used to predict the clinical pregnancy outcome in patients who have undergone IVF/intracytoplasmic sperm injection (ICSI) cycles. In the present study, the data of 512 infertile couples undergoing fresh IVF treatment in the Reproductive Medicine Center of our hospital between 11/2019 and 08/2021 were retrospectively studied. We investigated the relationship between the clinical pregnancy rate after Day 3 or Day 5 fresh embryo transfer and the available blastocyst formation rate and provided information for the clinical use of in vitro fertilization-embryo transfer (IVF-ET) based on blastocyst culture.

## Materials and methods

### Patients and study design

This was a retrospective cohort study of women undergoing Day 3 or Day 5 fresh embryo transfer at the Department of Reproductive Medicine, Xiamen Maternity and Child Health Hospital from November 1, 2019, to August 31, 2021. Eligible patients were females younger than 35 years of age who were undergoing their first fresh IVF cycle using their own oocytes. The number of retrieved oocytes was no less than 5, and the proportion of mature oocytes (metaphase II oocytes, MII oocytes) on the day of oocyte recovery was ≥ 60%. Patients who had a history of recurrent pregnancy loss (RPL) (≥ 2 spontaneous abortions) or had underlying uterine malformations, chromosomal abnormalities, abnormal oocytes and cycles involving donor oocytes or embryos or severe male factors were excluded from the study. Patients were divided into two groups: the Day 3 transfer group (transfer after Day 3 and all the remaining cleavage embryos were cultured to the blastocyst stage) and the Day 5 transfer group (Day 5 blastocyst transfer was performed after all Day 3 cleavage embryos were cultured to the blastocyst stage). All data were extracted from our electronic medical record system; thus, informed consent was not needed.

### Embryo culture and assessment

Ovarian stimulation was carried out using standard protocols according to female age, basal hormone levels, basal ovarian reserve and body mass index (BMI). Ovulation was triggered mainly by human chorionic gonadotropin (hCG, AIzer, Switzerland Merck Serono) after dominant follicles reached a diameter of ≥ 18 mm, and oocyte retrieval was scheduled 36 h later under the guidance of vaginal ultrasound. IVF/ICSI was selected for insemination on the basis of semen quality. Oocyte maturity was assessed when granulosa cells were stripped after short-term fertilization in IVF or after hyaluronidase digestion in ICSI and the MII oocyte was observed for the presence of a polar body using an inverted microscope. Embryos were cultured individually in micro drops (25 μl) in IVF sequential culture medium (CM/BM media; COOK, Australia) throughout the entire developmental stage and incubated under mineral oil (Vitrolife, Sweden) in a low-oxygen atmosphere (6% CO_2_, 5% O_2_ and 89% N_2_) at 37 °C. Embryo morphology was assessed and recorded on Day 3 and Day 5 post-fertilization. Cleavage-stage embryos were evaluated on the basis of the cell number, symmetry, the fragmentation rate and the presence of multinucleated blastomeres [13–15]. According to the Istanbul consensus [16], a high-quality embryo on Day 3 was defined as follows: 7–9 blastomeres with less than 15% fragmentation and no vacuoles or multinucleation. An available day 3 embryo was defined as 6–12 blastomeres with less than 30% fragmentation. Blastocysts were assessed according to the Gardner and Schoolcraft blastocyst scoring system [17, 18], which was based on blastocyst expansion grades from 1 to 6, the number and cohesiveness of the ICM and TE organization scores of A, B, or C. Blastocyst outcomes (transfer, freezing and discarding) were based on morphological parameters. Available blastocysts were defined as those that met the following criteria: the blastocyst expanded up to 3 stages (cavity completely filling the embryo), and the ICM and the TE were scored as AA, AB, BA, BB, AC and BC. The remaining blastocysts were excluded from this study.

The MII oocytes rate was calculated as the total number of MII oocytes on the day of oocyte retrieval/the total number of retrieved oocytes×100%.

The 2PN oocytes rate in IVF was calculated as the total number of 2PN zygotes/the total number of retrieved oocytes×100%.

The 2PN oocytes rate in ICSI was calculated as the total number of 2PN zygotes/the total number of MII oocytes×100%.

The blastocyst formation rate was calculated as the total number of blastocysts formed on Day 5 and Day 6/the number of embryos that underwent blastocyst culture at Day 3 × 100%.

The high-quality embryo rate for transplantation was calculated as the total number of high-quality embryos transferred/the total number of embryos transferred × 100%.

The available blastocyst formation rate was calculated as the total number of available blastocysts on Day 5 and Day 6/the number of embryos that underwent blastocyst culture at Day 3 × 100%.

The Day 5 available blastocyst formation rate was calculated as the total number of available blastocysts on Day 5/the number of embryos that underwent blastocyst culture at Day 3 × 100%.

### Embryo transfer and clinical outcome

All embryo transfers were performed under the guidance of abdominal ultrasound, and 90 mg/d vaginal progesterone sustained-release vaginal gel (Snoton, Merck Serono) was administered for luteal support immediately after transfer. Serum β-HCG was measured on the 14th day after embryo transfer. The outcome of the study was the clinical pregnancy rate (CPR), and clinical pregnancy was confirmed by the visualization of a gestational sac on transvaginal ultrasound scan.

### Statistical analysis

SPSS 25.0 software was used for statistical analysis. Comparisons were made using Chi-square or Fisher’s exact tests for categorical variables. Continuous variables are expressed as the mean ± standard deviation (SD) or median (P25, P75), which was based on the data distribution. All tests were two-sided, and a *P* value < 0.05 indicated that the differences were statistically significant.

## Results

**Fig. 1:**
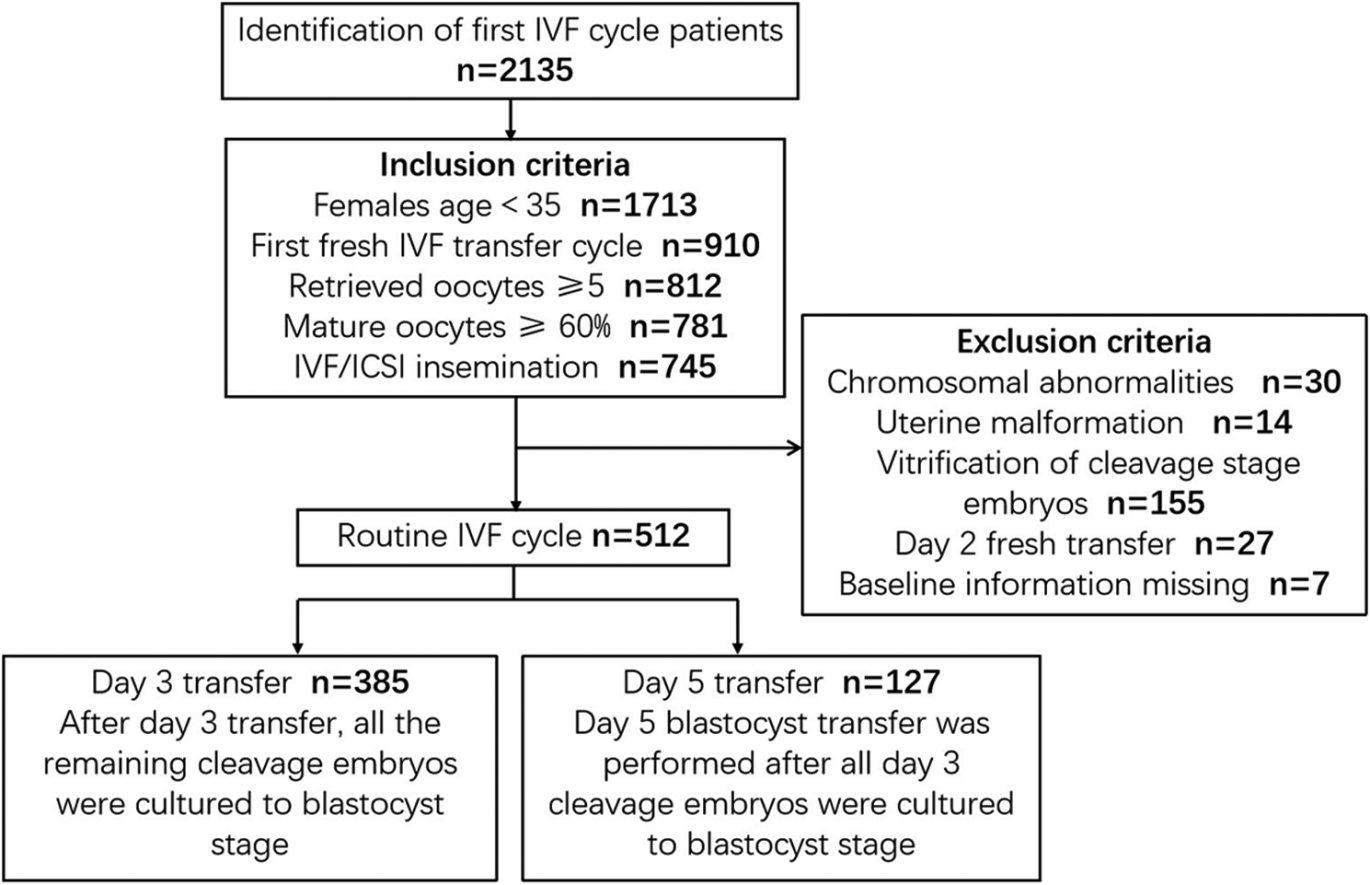
Study design flow diagram. First, IVF cycle patients who met the inclusion criteria were included in the study, and patients who met the exclusion criteria were excluded. According to the embryo development days, eligible patients who underwent routine fresh IVF cycles were divided into Day 3 and Day 5 transfer groups. The baseline characteristics and laboratory data of the two groups were compared according to the clinical pregnancy outcome

After exclusions, a total of 512 women were included for analysis. Figure 1 depicts the flow of study participants. The baseline characteristics and laboratory data of these couples are summarized in Tables 1 and 2, respectively.

The baseline characteristics and laboratory data of couples with Day 3 transfer are shown in Table 1. Compared with the nonclinical pregnancy group, the clinical pregnancy group had a higher 2PN oocyte rate (69.32% vs. 66.05%, *P* = 0.041), a higher available embryo rate at Day 3 (82.44% vs. 78.49%, *P* = 0.016) and more embryos transferred (*P* = 0.016). The ovulation promotion regimen between the two groups had significant difference, the clinical pregnancy group used GnRH agonist protocol more often (*P* = 0.002, see Supplementary Table 1); the blastocyst formation rate and available blastocyst formation rate showed no significant differences between the clinical pregnancy group and nonclinical pregnancy group (*P* > 0.05). The infertility causes, high-quality embryo rate for transplantation and other remaining results the remaining results were also not significantly different (*P* > 0.05). These results suggested that the remaining cleavage embryos with comparative development potential to reach the blastocyst stage between the two groups with Day 3 transfer and Day 3 transfer with two fresh available embryos could improve the clinical pregnancy rate.

**Table 1:** The baseline characteristics and laboratory data of the Day 3 transfer (women with clinical pregnancy) and control groups

	Clinical Pregnancy	No Clinical Pregnancy	*P* value
Number of patients	275	110	
Female Age (years)	29.71 ± 2.74	30.02 ± 2.71	0.322
Male Age (years)	31.31 ± 3.58	31.34 ± 3.50	0.953
Female BMI (kg/m^2^)	21.55 ± 2.51	21.43 ± 2.38	0.647
Infertility duration (years)	3.34 ± 2.14	3.28 ± 2.13	0.776
bFSH	7.72 ± 2.39	7.41 ± 2.06	0.229
Gn dosage (IU/L)	2350.32 ± 650.17	2351.36 ± 615.55	0.988
Oocytes retrieved (n) Fertilization model (%)	11.05 ± 3.94	11.04 ± 4.15	0.974 0.899
IVF	226 (82.2)	91 (82.7)	
ICSI	49 (17. 2)	19 (17.3)	
Endometrial thickness on ET day (cm)	11.76 ± 6.28	11.04 ± 2.11	0.240
No. of embryos transferred, n (%)			0.016*
1	21/529 (4.0)	17/203 (8.4)	
2	508/529 (96.0)	186/203 (91.6)	
MII oocytes rate (%)	88.48% (2689/3039)	87.81% (1066/1214)	0.537
2PN oocytes rate (%)	69.32% (2054/2963)	66.05% (782/1184)	0.041*
Cleavage rate of 2PN oocytes (%)	98.20% (2017/2054)	98.08% (767/782)	0.836
Available embryo rate at Day 3 (%)	82.44% (1663/2017)	78.49% (602/767)	0.016*
High-quality embryo rate at Day 3 (%)	27.12% (547/2107)	27.77% (213/767)	0.331
Blastocyst formation rate (%)	66.71% (1118/1676)	66.51% (419/630)	0.928
High-quality embryo rate for transplantation	41.97% (222/529)	40.39% (82/203)	0.699
Available blastocyst formation rate (%)	48.45% (812/1676)	48.73% (307/630)	0.904
Day 5 available blastocyst formation rate (%)	33.29% (558/1676)	35.08% (221/630)	0.419

Note: Values are presented as the mean ± standard deviation (SD) or number (%)

BMI: Body mass index

bFSH: Basal follicle-stimulating hormone

Gn: Gonadotropin

*P < 0.05 was considered statistically significant.

The baseline characteristics and laboratory data of the Day 5 transfer group are shown in Table 2. Compared with the nonclinical pregnancy group, the clinical pregnancy group had a lower blastocyst formation rate, but the difference was not statistically significant (77.03% vs. 81.06%, *P* = 0.083). Similarly, women with clinical pregnancy had a lower available blastocyst formation rate, and the difference was statistically significant (60.79% vs. 66.19%, *P* = 0.014). Also, the Day 5 available blastocyst formation rate in the clinical pregnancy groups was statistically significant lower than the nonclinical pregnancy group (46.98% vs. 54.58%, *P* = 0.007). The infertility causes, ovulation promotion regimen, high-quality embryo rate for transplantation and other remaining results showed no significant differences between the two groups (*P* > 0.05, see Supplementary Table 2).

**Table 2:** The baseline characteristics and laboratory data of the Day 5 transfer (women with clinical pregnancy) and control groups

	Clinical Pregnancy	No Clinical Pregnancy	*P* value
Number of patients	81	46	
Female Age (years)	29.33 ± 2.69	29.93 ± 2.71	0.230
Male Age (years)	30.86 ± 3.23	31.93 ± 4.25	0.113
Female BMI (kg/m^2^)	21.65 ± 2.63	21.55 ± 3.04	0.857
Infertility duration (years)	3.50 ± 1.96	3.33 ± 2.55	0.676
bFSH	7.53 ± 2.77	8.15 ± 4.04	0.305
Gn dosage (IU/L)	2251.24 ± 545.51	2281.79 ± 699.25	0.799
Oocytes retrieved (n) Fertilization mode (%)	13.59 ± 3.48	13.39 ± 3.44	0.753 0.255
IVF	74 (91.4)	39 (84.8)	
ICSI	7 (8.6)	7 (15.2)	
Endometrial thickness on ET day (cm)	11.57 ± 2.12	11.76 ± 1.97	0.629
No. of embryos transferred, n (%)			0.745
1	80/82 (97.6)	46/46 (100.0)	
2	2/82 (2.4)	0	
MII oocytes rate (%)	92.01% (1013/1101)	93.18% (574/616)	0.378
2PN oocytes rate (%)	74.24% (804/1083)	76.19% (464/609)	0.374
Cleavage rate of 2PN oocytes (%)	98.89% (795/804)	98.92% (459/464)	0.945
Available embryo rate at Day 3 (%)	89.69% (713/795)	88.02% (404/459)	0.362
High-quality embryo rate at Day 3 (%)	41.89% (333/795)	42.48% (195/459)	0.837
Blastocyst formation rate (%)	77.03% (664/862)	81.06% (398/491)	0.083
High-quality embryo rate for transplantation	58.54% (48/82)	69.57% (32/46)	0.216
Available blastocyst formation rate (%)	60.79% (524/862)	66.19% (325/491)	0.014*
Day 5 available blastocyst formation rate (%)	46.98% (405/862)	54.58% (268/491)	0.007*

Note: The blastocyst formation rate was calculated as the total number of blastocysts formed on Day 5 and Day 6/the number of embryos that underwent blastocyst culture at Day 3 × 100%

Values are presented as the mean ± standard deviation (SD) or number (%)

BMI: Body mass index

bFSH: Basal follicle-stimulating hormone

Gn: Gonadotropin

*P < 0.05 was considered statistically significant.

**Fig. 2:**
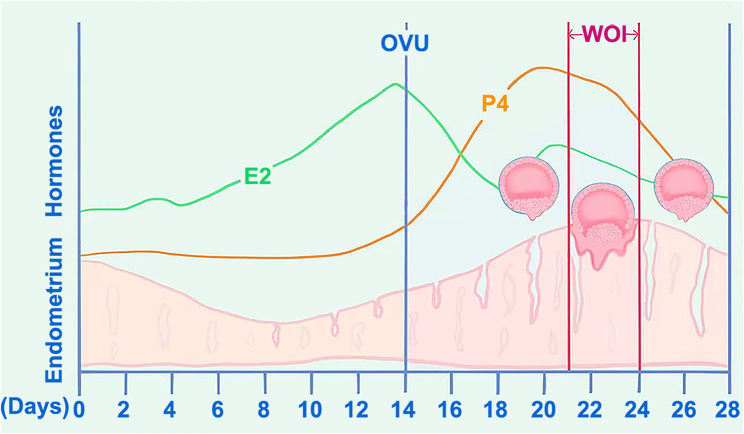
Days of the menstrual cycle and the blastocyst WOI. Implantation is a complex process involving both the blastocyst and the maternal endometrium, and their interaction can only occur during a limited defined period, known as the WOI. Well-developed blastocysts adhere before or after the WOI and then miss the WOI. Note: E2: Estradiol, OVU: Ovulation, P4: Progesterone, WOI: window of implantation

## Discussion

Cleavage-stage embryo transfer is often carried out in certain patients [2], such as in those cleavage-stage embryos with a lower ability to develop into available blastocysts to reduce the risk of cycle cancellation. Beyond that, to overcome deficiencies in embryo viability assessment, many IVF centers worldwide choose to transfer more than one embryo in one cycle [19]. In this study, in the Day 3 transfer group, compared with the nonclinical pregnancy group, the clinical pregnancy group had a higher 2PN oocyte rate and available embryo rate at Day 3, but the Day 3 high-quality embryo rate, blastocyst formation rate and available blastocyst formation rate showed almost no difference between the two groups. The reason for these results may be that the clinical pregnancy group had more Day 3 fresh available embryos for transplantation than the control group.

An available blastocyst is defined as the presence of a new good or excellent quality, expanded, hatching, or hatched blastocyst [20]. The available blastocyst formation rate refers to the percentage of embryos that reach the blastocyst stage and are suitable for transfer or freezing, it depends on patient population, ovarian stimulation, cell culture and criteria used for selection and so on [21, 22]. In the present study, the available blastocyst formation rate in the Day 5 transfer group differed between clinical pregnancy and nonclinical pregnancy groups (60.79% vs. 66.19%, *P* = 0.014), it was significantly higher in the nonclinical group than that in the clinical pregnancy group. And the Day 5 available blastocyst formation rate showed the same pattern between clinical pregnancy and nonclinical pregnancy groups (46.98% vs. 54.58%, *P* = 0.007). In the 5-year multicenter, international, randomized controlled trial (RCT), Carlos et al. [23] evaluated personalized embryo transfer (PET) guided by the endometrial receptivity analysis (ERA) test versus frozen embryo transfer (FET) or fresh embryo transfer, pregnancy rates at the first embryo transfer in PET, FET and fresh embryo transfer arms were 72.5% versus 54.3% (*P* = 0.01) and 58.5% (*P* = 0.05), respectively, while the blastocyst development rates (51.9%, 51.3% and 51.3%, *P* > 0.05) were similar in three groups. Similarly, Gardner et al. [24]. indicated that irrespective of the number of blastocysts formed, pregnancy rates were similar. The study by Roelens et al. [25] suggested that some patients didn’t conceive after embryo transfer (ET) due to suboptimal timing rather than a pathologic issue. Meanwhile, one additional day of P treatment led to an increase in the live birth rate for the slower developing blastocyst embryos [26]. By considering the developmental stage of the blastocyst and tailoring endometrial preparation optimally, patients might experience higher live birth rates.

The high percentage of available blastocysts indicated that the overall embryo developmental potential was good, but IVF treatments were not successful when high-quality embryos or even euploid embryos were transferred into the endometrial cavity, which may be due to endometrial factors [27, 28]. It is well known that implantation is a critical step in human reproduction [9, 11]. The success of ET relies on synchronization between the embryo and endometrium so that the endometrium is optimally receptive embryo implantation [9, 29]. Previous studies had investigated that women with repeated implantation failure (RIF), and personalized transfer timing resulted in a higher clinical pregnancy rate compared with routine protocols [30–32]. Thus, although there is a benefit favoring blastocyst transfer in fresh cycles, it remains unclear whether the day of transfer impacts the pregnancy rate [4]. Carlos suggested statistically significant improvement in the pregnancy, implantation and cumulative live birth rates in the PET arm compared with FET and fresh embryo transfer arms, indicating the potential utility of PET guided by an ERA test at the first appointment [23].

In humans, the WOI corresponds to the mid-secretory phase, occurring between the 20th and 24th day of the menstrual cycle or 6–10 days after the luteinizing hormone (LH) peak [33] (Fig. 2). However, embryos with low-speed growth rate are intrinsically different and require a longer duration of progesterone exposure for optimal synchronization with the endometrium [25]. We postulated that in our study, well-developed blastocysts adhered before or after the WOI and then missed the WOI (Fig. 2). Overall, embryo quality was a key factor in determining pregnancy, and other factors, including a receptive endometrium, were also considered to be predictive. High-quality embryos and the appropriate endometrial preparation protocol both had great significance for improving the ET pregnancy rate. Thus, further study is warranted to explore personalized treatment regarding transfer timing to improve pregnancy outcomes or with whole embryo freezing in ET cycles.

In addition to embryonic development potential, progesterone and estrogen levels are indeed important factors that can affect clinical pregnancy rates in IVF [34], and abnormal progesterone levels prior to transfer were associated with live-birth rates (LBR) and miscarriage rates (MR) after PGT-A [35]. Hormonal supplementation is commonly provided to ensure appropriate levels of progesterone and estrogen. The fertility medical professionals evaluate the patients’ specific situation, monitor their hormone levels during the ET cycles, which consistent with a receptive endometrium, and adjust the medication doses as needed to optimize the chances of a successful pregnancy. So, in the present study the hormone levels of estrogen and pregnancy were not related, future research should be pay more attention to progesterone levels prior to transfer. Infertility factors, ovulation promotion regimen are also important factors that can affect clinical pregnancy rates in IVF. Although from the limited data, the comparison between the different infertility causes were no difference, regardless of each couple’s nature infertility causes, it resulted in similar outcomes.

The strength of the study was that it was the first time to find that the available blastocyst formation rate was not positively correlated with clinical pregnancy outcome in the Day 5 fresh blastocyst transfer group. Embryo selection aims at shortening the time-to-pregnancy, while minimizing the reproductive risks. Knowing which features are associated with the reproductive competence of blastocysts is therefore critical to define, implement, and validate safer and more efficient clinical work. So, a good clinical pregnancy outcome requires not only embryos with good developmental potential, but also a receptive endometrium and synchronization between the two, future researches should be directed to improve evaluation of the blastocyst-endometrial dialogue. At the same time, we acknowledged that our study had limitations, its small sample size and the retrospective nature of the analysis still needed further researches and studies.

In conclusion, our results suggested that the available blastocyst formation rate was not associated with the clinical pregnancy outcome of patients undergoing Day 3 fresh embryo transfer, and the available blastocyst formation rate was not positively correlated with clinical pregnancy outcome for the Day 5 fresh blastocyst transfer. Based on these findings, we further confirmed that for a successful clinical pregnancy in artificial cycles with women undergoing fresh embryo transfer, a competent blastocyst synchronized with a receptive endometrium was needed, which indicated that more work should to improve clinical implantation by personalizing, diagnosing and synchronizing endometrial factors.

### Electronic supplementary material

Below is the link to the electronic supplementary material.

Supplementary Material 1

## Data Availability

The datasets used and/or analysed during the current study are available from the corresponding author on reasonable request.
