# Supplementary material for: Competent blastocyst and receptivity endometrium improved clinical pregnancy in fresh embryo transfer cycles: a retrospective cohort study
Source: BMC Pregnancy Childbirth. 2024 Apr 11;24:258. doi: 10.1186/s12884-024-06399-x (PMC11007979; doi:10.1186/s12884-024-06399-x)
Supplement: Supplementary file 1 — Supplementary Material 1 [file 12884_2024_6399_MOESM1_ESM.docx]

**Table 1** The baseline characteristics and laboratory data of the Day 3 transfer (women with clinical pregnancy) and control groups

|  | Clinical Pregnancy | No Clinical Pregnancy | *P* value |
| --- | --- | --- | --- |
| Number of patients | 275 | 110 |  |
| Infertility causes, n (%) |  |  | 0.370 |
| Ovulation dysfunction | 31 (11.3) | 7 (6.4) |  |
| Pelvic and fallopian tube factors | 146 (53.1) | 70 (63.6) |  |
| Endometriosis | 35 (12.7) | 12 (10.9) |  |
| Male factor | 34 (12.4) | 12 (10.9) |  |
| Unexplained | 29 (10.5) | 9 (8.2) |  |
| Ovulation promotion regimen, n (%) |  |  | **0.002*** |
| GnRH agonist protocol | 258 (93.8) | 92 (83.6) |  |
| GnRH antagonist protocol | 17 (6.2) | 18 (16.4) |  |

**Table 2** The baseline characteristics and laboratory data of the Day 5 transfer (women with clinical pregnancy) and control groups

|  | Clinical Pregnancy | No Clinical Pregnancy | *P* value |
| --- | --- | --- | --- |
| Number of patients | 81 | 46 |  |
| Infertility causes, n (%) |  |  | 0.534 |
| Ovulation dysfunction | 8 (9.9) | 6 (13.0) |  |
| Pelvic and fallopian tube factors | 57 (70.3) | 28 (60.9) |  |
| Endometriosis | 8 (9.9) | 5 (10.9) |  |
| Male factor | 3 (3.7) | 5 (10.9) |  |
| Unexplained | 5 (6.2) | 2 (4.3) |  |
| Ovulation promotion regimen, n (%) |  |  | 1.000 |
| GnRH agonist protocol | 77 (95.1) | 44 (95.7) |  |
| GnRH antagonist protocol | 4 (4.9) | 2 (4.3) |  |
